# Temporal changes in rabies epidemiology in Brazil: an analysis of notifications of suspected and confirmed human rabies cases, 2010-2025

**DOI:** 10.1590/S1678-9946202668060

**Published:** 2026-09-18

**Authors:** João Vitor Engelhardt, Halana do Carmo Silva, Gabriel Campana do Nascimento, Arthur Moraes Lavinas, Beatriz Biancardi de Campos, Eduarda Scárdua Coser, Bruno Vidal Boscaglia, Elinara Ferreira Santos, Sarah Fernandes Teixeira, Mariana Rambaldi do Nascimento, Joamyr Victor Rossoni, Clairton Marcolongo-Pereira

**Affiliations:** 1 Centro Universitário do Espírito Santo, Faculdade de Medicina, Programa de Pós-Graduação em Ciências da Saúde, Colatina, Espírito Santo, Brazil; 2 Centro Universitário do Espírito Santo, Faculdade de Medicina, Colatina, Espírito Santo, Brazil

**Keywords:** Human rabies, Desmodus rotundus, Sylvatic cycle, Epidemiology, Antigenic variant

## Abstract

Human rabies is an invariably fatal encephalitis caused by *Lyssavirus* species, with near-100% lethality after symptom onset. In Brazil, canine vaccination has markedly reduced urban transmission. However, human deaths continue to occur and are increasingly linked to sylvatic reservoirs, particularly *Desmodus rotundus* bats. This study describes the epidemiological profile of human rabies in Brazil from 2010 to 2025, focusing on temporal trends, aggressor species, demographics, geographic distribution, and genetic/antigenic variants. This descriptive time-series study used secondary data from the Notifiable Diseases Information System (SINAN/DATASUS): 1,482 notifications from 16 database files. Antigenic variant data were obtained from the Ministry of Health, and spatial distribution was analyzed using QGIS. Of these, 46 (3.1%) were confirmed, and 41 had recorded dates of death in SINAN. Peaks in the notifications of suspected rabies cases occurred in 2017 (n = 228), whereas confirmed cases peaked in 2018 (n = 11), when all infections were attributed to bats, including a cluster of 10 cases in Para. Men accounted for 55.9% of the notifications, and those aged ≥ 60 and 30-39 years were the most affected. Dogs caused 65.6% of the aggressions, but bats predominated among confirmed cases from 2017 onward. Genetic/antigenic variant analysis identified three active sylvatic cycles: AgV3 (*D. rotundus*), marmoset lineage (*Callithrix* *jacchus*), and wild canid lineage (*Cerdocyon thous*). No confirmed human rabies cases associated with domestic canine lineages or variants have been reported since 2015. These findings show a shift from urban dog-mediated rabies to sylvatic rabies in Brazil.

## INTRODUCTION

Rabies is an acute encephalitis that is almost invariably fatal after the onset of clinical symptoms. Rabies is caused by viruses of the genus *Lyssavirus*, which are transmitted to humans via saliva or exposure to nervous tissue of infected animals, most commonly via bites^1^. Rabies, one of the oldest known zoonoses, remains a major global public health concern, responsible for approximately 59,000 deaths annually across 150 countries, with 95% of cases occurring in Asia and Africa^2^ ^,^ ^3^. Although this estimate is based on modelling data generated more than a decade ago, it remains the most recent global estimate reported by the World Health Organization^3^. The disease progresses with a variable incubation period and presents in two main clinical forms: encephalitic (“furious”) rabies, characterized by hyperactivity, hydrophobia, and aerophobia, and paralytic rabies, marked by progressive flaccid weakness^1^. Once neurological symptoms appear, the case fatality rate approaches 100%, making rabies one of the few infectious diseases for which a clinical cure remains exceptional^4^.

Despite its lethality, rabies is entirely preventable. Post-exposure prophylaxis (PEP), which involves immediate wound cleaning, vaccination, and rabies immunoglobulin administration when indicated, is highly effective when initiated promptly after exposure^5^. Each year, more than 29 million people worldwide receive post-bite vaccination, preventing an estimated 10,000 deaths^3^. However, barriers, including delays in seeking care, incomplete prophylactic regimens, and limited healthcare infrastructure, continue to compromise PEP effectiveness in endemic regions^5^ ^,^ ^6^.

In Brazil, rabies is a notifiable disease under the national epidemiological surveillance system. Historically, domestic dogs have been the primary source of human infections, driving the establishment of large-scale national programs for canine vaccination and human prophylaxis^7^ ^,^ ^8^. These efforts have dramatically reduced urban cycle transmission, from 50 dog-mediated human deaths in 1990 to zero confirmed domestic canine-variant cases since 2015^9^. However, this achievement has unmasked the growing epidemiological relevance of the sylvatic cycle, particularly transmission by hematophagous vampire bats (*Desmodus rotundus*), which maintain independent enzootic circulation of rabies virus in rural and forested areas across the country^9^ ^,^ ^10^.

This transition in transmission dynamics poses fundamentally different challenges for surveillance and control. Unlike domestic dogs, bat populations cannot be vaccinated at scale, and the communities most exposed to vampire bat predation, rural settlements in the Amazon and northeastern Brazil, are frequently those with the least access to timely PEP^11^ ^,^ ^12^. Despite the public health significance of this shift, its characterization has largely relied on aggregate data from the Ministry of Health bulletins, with limited analysis of individual-level notification records across the entire spectrum of epidemiological variables.

Therefore, this study aims to describe the epidemiological profile of human rabies notifications and confirmed cases in Brazil from 2010 to 2025, using individual-level data from the Information System for Notifiable Diseases (SINAN) and focusing on finding the temporal shift in aggressor species, demographic characteristics of affected individuals, geographic distribution of confirmed cases across Brazilian states, and the implications for public health surveillance.

### Ethics

This study was exempt from ethical review in accordance with Resolution no. 510/2016 of the Brazilian National Health Council because it used publicly available, anonymized, and aggregated secondary data.

## MATERIALS AND METHODS

This descriptive observational time-series study was based on secondary data from the Information System for Notifiable Diseases (SINAN), maintained by the Brazilian Ministry of Health and made publicly available by the Department of Informatics of the Unified Health System (DATASUS)^13^. In the Brazilian surveillance system, a rabies notification corresponds to any suspected case that meets the national case definition, which must be reported within 24 h of suspicion, regardless of subsequent laboratory confirmation^14^. All human rabies suspected notifications (ICD-10: A82.9) meeting this definition and recorded in Brazil from January 1, 2010, to December 31, 2025, were included, totaling 1,482 records distributed across 16 compressed database files (DBC format).

Data were extracted and processed in Python (version 3.10). The pyreaddbc library was used to decompress and convert DBC files to a readable tabular format, and the pandas library, to organize and analyze data. The following variables were analyzed: year of notification, state of notification, state of residence, sex, age group, race/skin color, educational level, aggressor species, type of exposure (bite, scratch, mucosal contact, or licking), vaccination status of the aggressor animal, hospitalization, final case classification (confirmed, discarded, or inconclusive), and death, identified by the presence of a recorded date in the death field (DT_OBITO). Death was assessed solely by the presence of a recorded date in this field. Thus, missing death dates were not interpreted as evidence of survival.

Within the SINAN classification framework, a notified case refers to any report registered in the system after meeting the definition of a suspected case. A suspected case is defined as an individual presenting a clinical picture compatible with rabies and/or a history of exposure (bite, scratch, or mucosal/cutaneous contact with saliva or nervous tissue) to an animal suspected or confirmed to be rabid, regardless of laboratory outcome. Following clinical and epidemiological investigation, each notified case is assigned a final classification (confirmed, discarded, or inconclusive). A case is classified as confirmed when laboratory criteria are met (direct fluorescent antibody testing, virus isolation, reverse transcription polymerase chain reaction, or post-mortem histopathological detection of Negri bodies) or, in the absence of conclusive laboratory testing, when clinical-epidemiological criteria are met, defined as a compatible clinical presentation associated with documented exposure to a confirmed or highly suspected rabid animal. A case was classified as discarded when laboratory testing was negative or when the clinical-epidemiological criteria for rabies were unmet. Cases were classified as inconclusive when the final outcome was impossible to be determined, most commonly due to death before sample collection, loss to follow-up, or incomplete investigation^14^.

Age was decoded from the SINAN-specific numeric format (NU_IDADE_N), in which values ≥ 4000 represent age in years, values ≥ 3000 represent age in months, and values ≥ 2000 represent age in days. Age groups were defined as: 0-4, 5-9, 10-14, 15-19, 20-29, 30-39, 40-49, 50-59, and 60 years or older. Categorical variables were described by absolute and relative frequencies, and the temporal distribution of notifications and confirmed cases was analyzed by year. The aggressor species was analyzed for the overall set of notifications and exclusively for confirmed cases to identify shifts in transmission patterns over time. A dedicated demographic and epidemiological analysis (sex, age, geographic region, source of infection, and clinical outcome) was further conducted for the subset of 46 laboratory-confirmed cases, enabling direct comparisons with the demographic profile of all notifications. Statistical figures were generated using the Matplotlib library (Python 3.10), and the geographic distribution of notifications and confirmed cases by federative unit was visualized via thematic choropleth maps produced on QGIS (version 3.44.9 Solothurn), using a base map of Brazil provided by the Brazilian Institute of Geography and Statistics^15^.

Additionally, this demographic analysis of confirmed cases was complemented by case-level data on genetic lineages/antigenic variants from an official table published by the Health Surveillance and Environment Secretariat of the Brazilian Ministry of Health (updated January 2026)^16^. This source provided information on aggressor species and rabies virus characterization for confirmed human rabies cases recorded from 2010 to 2025, when such characterization was performed and available in official records. These records were cross-checked against the corresponding SINAN entries by year, state, and municipality to confirm the aggressor species classification and complete missing or non-specific species codes (e.g., generic “other” categories) for the subset of laboratory-confirmed cases. They were used to characterize the genetic lineages/antigenic variants associated with each transmission cycle and to contextualize the probable sylvatic origin of exposures involving domestic animals.

## RESULTS

From 2010 to 2025, SINAN recorded 1,482 notifications of suspected human rabies cases. Annual notification counts varied considerably over the period, ranging from seven cases in 2022 to 228 in 2017; 2016-2021 accounted for the highest volume, with 1,066 notifications (71.9% of the total) (Supplementary Table S1).

**Figure 1:**
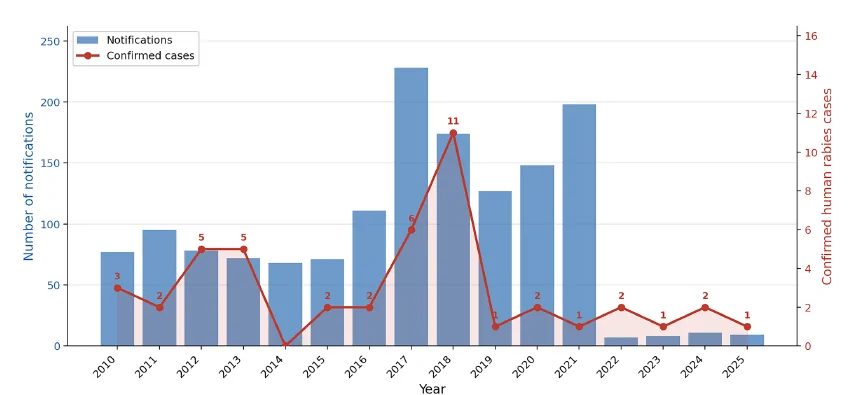
Annual human rabies notifications and confirmed cases in Brazil, 2010-2025. The bars represent the total number of notifications recorded in the Information System for Notifiable Diseases (SINAN), and the red line indicates the number of confirmed cases per year based on SINAN data. The numbers above the data points indicate the annual count of SINAN-confirmed cases (total n = 46).

Of the 1,482 notifications, SINAN classified 46 (3.1%) as confirmed human rabies cases; this number differs from the cumulative total of confirmed cases reported on the official webpage of the Ministry of Health for the same period (53 cases)^17^. The annual distribution of confirmed cases was irregular, with no confirmation in 2014. A peak of 11 confirmed cases occurred in 2018, the highest annual count in the study period. Among the 46 confirmed cases, 41 had recorded death dates (Figure 1 and Supplementary Table S1). The remaining five had no recorded death date in SINAN at the time of data extraction; of these, one corresponds to a documented survivor (Barcelos, Amazonas, 2017), recorded as the second documented case of human rabies survival in Brazil^18^.

Of all the notifications, 829 (55.9%) involved male individuals and 650 (43.8%), female ones; three records had sex recorded as unknown (Supplementary Table S2). Male predominance remained consistent across all analyzed years.

**Figure 2:**
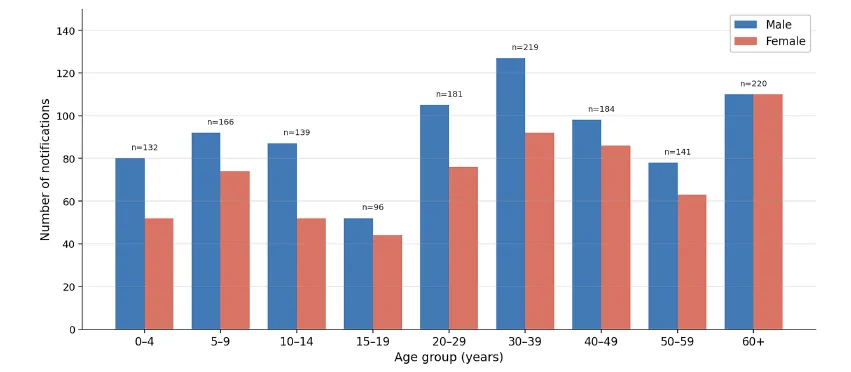
Human rabies notifications by age group and sex in Brazil, 2010-2025. Values above the bars indicate the total number of notifications per age group (both sexes combined).

Notifications were distributed across all age groups. Adults aged 60 years or older represented the largest single group (n = 220; 14.8%), followed by those aged 30-39 (n = 219; 14.8%), 40-49 (n = 185; 12.5%), and 20-29 (n = 182; 12.3 %) years. Among individuals aged < 15 years, the 5-9 age group had the highest number of notifications (n = 166; 11.2%), exceeding younger (0-4 years; n = 132; 8.9%) and older (10-14 years; n = 139; 9.4%) pediatric groups (Figure 2; Supplementary Tabl S2). Table 1 summarizes the sociodemographic and clinical characteristics of all notifications.

**Table 1:** Sociodemographic and clinical characteristics of human rabies notifications, Brazil, 2010-2025 (n = 1,482).

Characteristic	n	%
Ethnicity/skin color		
White	622	41.9
Mixed-race/Brown	595	40.1
Black	91	6.1
Asian	12	0.8
Indigenous	11	0.7
Unknown	122	8.2
Educational		
Missing/not applicable	546	36.8
Incomplete primary (1st-4th grade)	169	11.4
Complete secondary	136	9.2
Incomplete lower secondary (5th-8th grade)	125	8.4
Type of exposure		
Biting	907	61.2
Scratching	210	14.2
Aggressor animal vaccination status		
Unvaccinated	352	23.7
Vaccinated	293	19.8
Unknown	477	32.2
Hospitalization		
Not hospitalized	908	61.3
Hospitalized	186	12.5
Unknown	110	7.4
Final classification		
Discarded	876	59.1
Inconclusive	478	32.2
Confirmed	46	3.1
Unclassified	82	5.5

White and mixed-race/Brown individuals predominated, accounting for over 80% of the notifications. Educational level showed a high proportion of missing or non-applicable records (36.8%); among those with recorded data, incomplete primary schooling was the most frequent category. Biting was the most common type of exposure (61.2%), followed by scratching (14.2%). Most aggressor animals had unknown (32.2%) or unvaccinated (23.7%) vaccination status, and most individuals underwent no hospitalization (61.3%) (Table 1).

To enable comparisons against the overall notification profile described above, we conducted a dedicated demographic and epidemiological analysis restricted to the 46 laboratory-confirmed human rabies cases (Table 2). Confirmed cases predominantly involved men and younger age groups, with the Northeast and North regions accounting for over 80% of the cases. Bats were the leading source of infection, and AgV3 was the predominant antigenic variant, followed by AgV2 (canine cycle) and the marmoset-associated lineage. The clinical outcome was fatal in most cases; one case was documented as a survivor, whereas outcome data were unavailable for four cases, representing a data gap rather than confirmed survival.

**Table 2:** Demographic, epidemiological, and virological characteristics of confirmed human rabies cases in Brazil, 2010-2025 (n = 46).

Characteristic	n	%
Sex		
Male	34	73.9
Female	12	26.1
Age group (years)		
0-9	12	26.1
10-19	10	21.7
20-39	15	32.6
40-59	8	17.4
≥ 60	1	2.2
Median age (range), years	22.5 (1-67)	-
Geographic region		
Northeast	22	47.8
North	16	34.8
Southeast	4	8.7
Midwest	3	6.5
South	1	2.2
Reported aggressor species		
Bat	21	45.7
Dog	10	21.7
Non-human primate (marmoset)	7	15.2
Cat	4	8.7
Fox	2	4.3
Unidentified	2	4.3
Antigenic variant or genetic lineage		
AgV3	26	56.5
AgV2	8	17.4
Marmoset-associated genetic lineage	7	15.2
Wild canid-associated genetic lineage	2	4.3
AgV1	1	2.2
Not performed or not determined	2	4.3
Recorded outcome in SINAN		
Deaths recorded	41	89.1
Survivals recorded	1	2.2
Outcome not recorded	4	8.7

AgV = antigenic variant; SINAN = Notifiable Diseases Information System.

Notifications occurred in all Brazilian regions. The states with the highest notification counts were Sao Paulo (n = 325; 21.9%), Goias (n = 192; 12.9%), and Minas Gerais (n = 143; 9.6%), followed by Mato Grosso (n = 114; 7.7%), Paraiba (n = 92; 6.2%), Mato Grosso do Sul (n = 80; 5.4%), and Espirito Santo (n = 68; 4.6%).

**Figure 3:**
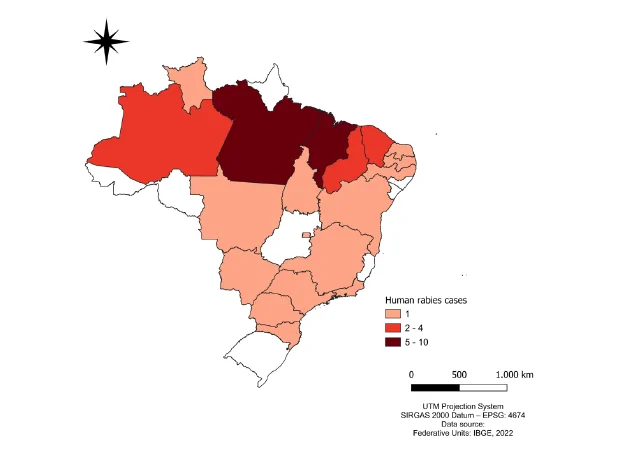
Choropleth map of confirmed human rabies cases by federative unit, Brazil, 2010-2025. Color intensity reflects the total number of confirmed cases, ranging from white (no confirmed cases) to dark red (highest number of cases).

The geographic distribution of confirmed rabies cases differed substantially from that of the notifications. States in the Brazilian North and Northeast, including Para, Maranhao, Amazonas, and Paraiba, accounted for a significant share of confirmed cases (Figure 3).

**Figure 4:**
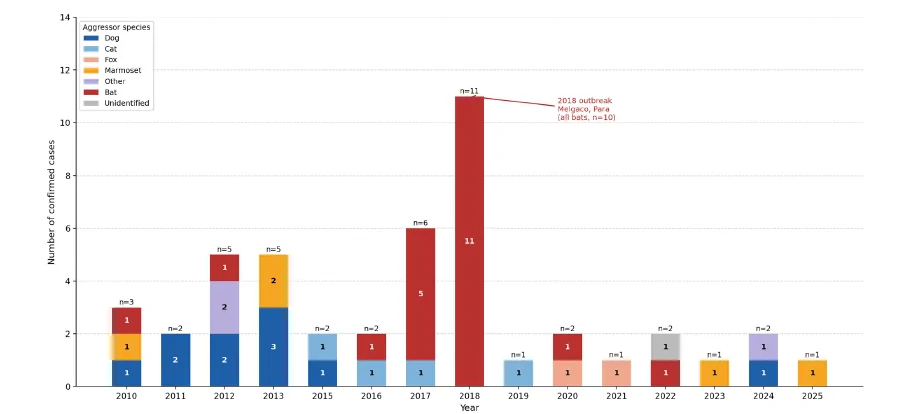
Confirmed human rabies cases by aggressor species and year, Brazil, 2010-2025. The stacked bars represent the number of SINAN-confirmed cases attributed to each species annually. The 2018 cluster in Melgaco, Para, constituted a recognized outbreak entirely attributed to exposure to bats (*Desmodus rotundus*). Numbers within segments indicate case counts, and values above the bars indicate annual totals based on SINAN data (total n = 46).

Of the 1,482 notifications, 1,204 (81.2%) contained information on aggressor species. Domestic dogs were the most frequent aggressors, followed by cats, bats, nonhuman primates (including marmosets), foxes, and domestic herbivores, with a substantial proportion recorded as unspecified or unknown (Supplementary Figure S1). However, a disaggregated analysis by year showed a significant and progressive shift in the predominant aggressor species in confirmed cases throughout the study period. From 2010 to 2015, domestic dogs were the primary aggressors in confirmed cases, consistent with the urban-transmission cycle. From 2017 onward, bats emerged as the dominant aggressors; of the six confirmed cases in 2017, five (83.3%) were attributed to bat exposure. In 2018, the year with the highest number of confirmed cases in the period, all 11 confirmed cases (100%) involved bat exposure, constituting a recognized outbreak in the municipality of Melgaco, Para, Brazil. In subsequent years, dog-transmitted confirmed cases became virtually absent, whereas bats and other sylvatic species continued to account for the rare confirmed cases recorded (Figure 4).

Complementary data from the Brazilian Ministry of Health^16^ provided genetic lineage/antigenic variant characterization for all confirmed human rabies cases from 2010 to 2025. The antigenic variant AgV3 was the most frequently identified across the study period, accounting for most confirmed cases since 2016. AgV3 was detected in cases directly attributed to bat exposure and in cases involving cats, bovines, and dogs.

Canine antigenic variants AgV2 and AgV1 were only identified in the early years of the study period. AgV2, an antigenic variant historically compatible with domestic dogs (*Canis lupus familiaris*), was identified in dog- and cat-associated cases in Maranhao (2011) and in dog-associated cases in Maranhao and Piaui (2013), with no further cases detected thereafter. A single case compatible with AgV1 was recorded in 2015 in the Mato Grosso do Sul State.

No human rabies cases associated with canine genetic lineages or antigenic variants have been confirmed since 2015.

Moreover, two additional sylvatic genetic lineages were identified in parallel with bat cycles. The genetic lineage compatible with *Callithrix jacchus* (lineage marmoset) was responsible for cases in the Brazilian Northeast across multiple years, including cases in Pernambuco, Ceara, Paraiba, Piaui, and Maranhao, with the most recent confirmed case recorded in 2025. The genetic lineage compatible with wild canids (wild canid lineage) was identified in fox-attributed cases in Maranhao (2021) and Paraiba (2020), reflecting an independent terrestrial-sylvatic transmission cycle.

Notably, all other confirmed cat-associated cases for which genetic lineage/antigenic variant data were available, including cases in Santa Catarina (2019), Pernambuco (2017), Roraima (2016), and Paraiba (2015), were compatible with AgV3. Similarly, a dog-attributed case in Tocantins (2024) was compatible with AgV3.

Regarding the final classification of all notifications, most were discarded following epidemiological and/or laboratory investigations (n = 876; 59.1%). A substantial proportion of cases remained inconclusive (n = 478; 32.2%). Confirmed cases accounted for 46 notifications (3.1%), and 82 records (5.5%) had no classification.

## DISCUSSION

This study provides a comprehensive epidemiological characterization of notifications of suspected human rabies cases in Brazil from 2010 to 2025 based on individual-level data from SINAN. Its findings confirm and extend, with quantitative evidence, a pattern previously described in qualitative terms: the progressive displacement of the domestic dog as the primary source of fatal human rabies cases in Brazil, replaced by bats, particularly the hematophagous vampire bat *D. rotundus*, as the dominant aggressor in confirmed cases from 2017 onward^7^ ^,^ ^9^.

The overall volume of notifications of suspected rabies cases peaked in 2017 (n = 228), declining sharply from 2022 onward; this should not be interpreted as reflecting a true reduction in exposure events. The low notification counts in recent years most likely reflect delays in data entry and consolidation within SINAN, a well-recognized limitation of passive surveillance systems in Brazil^7^. The relative stability of confirmed cases during this period supports this interpretation.

The predominance of male individuals among notifications (55.9%) is consistent with findings from previous Brazilian studies, being generally attributed to greater occupational and behavioral exposure to animals in outdoor and rural environments^7^ ^,^ ^8^. The broad age distribution, with adults aged 60 years or older and those aged 30-39 years representing the most notified groups, challenges the historical perception of rabies as a disease that predominantly affects children and young adults. While pediatric exposure remains significant, as evidenced by the 5-9 age group ranking highest among those under 15 years of age, the data suggest that the risk is distributed across all life stages, reflecting the widespread and varied nature of human-animal contact in Brazil.

The demographic profiles of laboratory-confirmed cases diverged from those of all notifications in clinically relevant ways. The predominance of bat-transmitted AgV3-associated cases in the North and Northeast regions among confirmed cases-exceeding the proportion in the overall notification profile-likely reflects the established enzootic cycle of *D. rotundus* in these regions and the underdiagnosis or underreporting of confirmed cases elsewhere in Brazil. Similarly, the concentration of confirmed cases in younger age groups is consistent with previous reports linking pediatric rabies fatalities to the delayed recognition of bat-bite exposure and incomplete PEP^19^. The near-uniform case fatality among confirmed cases, in contrast to the single documented survivor, underscores the continued near-100% lethality of clinical rabies in Brazil despite advances in intensive supportive care, reinforcing that pre- and post-exposure prophylaxis remain the only effective means of preventing death once after exposure.

The geographic concentration of notifications of suspected human rabies cases in Sao Paulo, Goias, and Minas Gerais likely reflects higher population density and stronger epidemiological surveillance capacity in these states rather than an inherently higher biological risk. In contrast, the distribution of confirmed cases points toward states in the North and Northeast of Brazil, such as Para, Maranhao, and Amazonas, which is consistent with the persistence of the sylvatic rabies cycle in regions showing forest fragmentation, extensive cattle ranching, and difficult access to PEP^11^ ^,^ ^12^.

The most epidemiologically significant finding of this study refers to the documented shift in aggressor species among the confirmed cases. From 2010 to 2015, dogs accounted for most confirmed rabies cases, reflecting the residual transmission from the urban cycle. Since 2017, bats have virtually become the sole source of confirmed fatal rabies, with five of six confirmed cases in 2017 and all 11 in 2018 being attributed to bat exposure. This pattern, observed in individual-level SINAN records extending to 2025, corroborates and quantifies the transition described by Horta *et al.* ^9^ using aggregate Ministry of Health data up to 2020. Our data confirm that this shift, rather than a transient phenomenon, constitutes a structural change in the transmission dynamics of human rabies in Brazil.

AgV3 is primarily associated with *D. rotundus*, but has also been identified in frugivorous bats of the genus *Artibeus* ^20^, reflecting its broad circulation within the aerial sylvatic cycle. Notably, cats constitute no reservoirs of rabies virus as they maintain neither a distinct genetic lineage nor antigenic variant associated with sustained viral circulation^21^. In contrast, dogs are considered primary reservoir species as they sustain well-defined, antigenic variants that support endemic transmission cycles^22^. The genetic lineage and antigenic variant data add critical depth to this interpretation. The documentation that all confirmed cat-associated cases carried AgV3, the antigenic variant compatible with *D. rotundus*, shows that reported feline transmissions, rather than representing an independent feline cycle, indicate spillover events from the bat cycle, with cats serving as incidental, intermediate hosts. The same applies to a case in 2024 that was attributed to a dog carrying AgV3. This has direct clinical implications: in areas of known vampire bat activity, cat or dog bites should prompt consideration of PEP regardless of the apparent vaccination status of the animal given the possibility of undetected bat exposure in the animal’s history.

Furthermore, genetic lineage and antigenic variant data show that bat-transmitted rabies in Brazil operates alongside at least two other transmission chains within the sylvatic cycle. The aerial sylvatic transmission chain associated with marmosets (*C. jacchus*), active throughout the Brazilian Northeast and producing confirmed cases as recently as 2025, and the terrestrial sylvatic transmission chain associated with the crab-eating fox (*Cerdocyon thous*)^23^, documented in 2020 and 2021, require distinct surveillance and response strategies. The coexistence of these transmission chains within the broader sylvatic cycle substantially increases the complexity of rabies elimination efforts in Brazil when compared to the single-cycle urban scenario that characterized the disease in previous decades.

The interruption of urban cycle transmission, confirmed by the absence of cases associated with canine antigenic variants after 2015, is an unequivocal public health achievement attributable to sustained canine vaccination programs. However, this success must not lead to complacency. Unlike domestic dogs, bat populations cannot be vaccinated on a large scale, and the lethal control of bat populations is neither ethically acceptable nor ecologically feasible under Brazilian law^9^. The decreasing volume of PEP doses administered in Brazil in recent years, as documented by Horta *et al*.^9^, raises concerns that access to timely prophylaxis may be deteriorating as bat-transmitted rabies becomes the dominant transmission pathway, a combination that warrants urgent policy attention.

Beyond the shift in transmission dynamics, the high proportion of inconclusive cases (32.2%) warrants careful attention. This figure likely reflects deaths occurring before sample collection could be completed for laboratory confirmation, logistical barriers to sample transport in remote regions, and heterogeneity in the diagnostic infrastructure across Brazilian states. Inconclusive cases may also reflect limitations related to data quality and completeness of the SINAN surveillance database. Reducing this proportion requires investments in decentralized laboratory capacity and training of local health teams in rabies case investigation protocols.

The near-universal lethality among confirmed cases (41 of 46 had a recorded death date in SINAN) is consistent with the well-established case fatality rate of human rabies, which approaches 100% once neurological symptoms appear^4^. Of the five cases without a recorded date of death, only one corresponded to a documented survivor. The absence of a recorded death date in the remaining four most likely reflects and incomplete report regarding the outcome field-a known limitation of SINAN as a passive surveillance system-rather than confirmed survival. The asymptomatic incubation period is the window of opportunity for survival, which may range from days to months depending on the site and severity of exposure; once neurological symptoms appear, survival is exceedingly rare^4^. This is illustrated by the documented survivor case in Barcelos, Amazonas State, in 2017^18^, who remained comatose for seven years before dying of pneumonia in 2025^18^ ^,^ ^24^. Even this exceptional outcome of intensive care management is associated with profound and lasting neurological sequelae.

This study collected no data on PEP administered to notified individuals as SINAN only records the vaccination status of the aggressor animal, ignoring the prophylaxis received by patients. More broadly, secondary data analyses fall subject to the inherent constraints of passive surveillance, including under-reporting, notification delays, and incomplete field records. The apparent reduction in notifications after 2021 is likely an artifact of data consolidation lag and should not be interpreted as a true epidemiological trend. The high proportion of missing data for variables such as educational level (36.8%) and animal vaccination status (32.2%) limits the ability to identify specific socioeconomic risk factors. The integration of genetic lineage/antigenic variant data from Health Surveillance and Environment Secretariat of the Brazilian Ministry of Health records partially addresses one limitation of SINAN-based studies. However, this supplementary dataset covers only confirmed cases, enabling no genetic lineage/antigenic variant-level analysis of the broader notification universe, including inconclusive and discarded records of the disease. Additionally, the number of confirmed human rabies cases identified by SINAN microdata analysis (46 cases, 2010-2025) differs from the cumulative total on the Ministry of Health’s official human rabies webpage (53 cases for the same period)^17^, most likely reflecting retrospective case reclassification after the data extraction for this study. As the official webpage undergoes continuous updates (whereas the SINAN microdata represent a fixed extraction), some divergence between these two sources is expected, reflecting an inherent limitation of working with notification-based surveillance data.

## CONCLUSION

Human rabies in Brazil has undergone a marked epidemiological transition, shifting from a predominantly urban dog-mediated disease to one increasingly driven by sylvatic transmission, particularly by *D. rotundus*. Although dogs remain responsible for most reported cases of aggression, confirmed human cases are primarily associated with wildlife reservoirs. The coexistence of three transmission chains within the sylvatic cycle associated with the antigenic variants AgV3 (bat) and the genetic lineages compatible with marmosets (*C. jacchus*) and wild canids highlight the increasing complexity of rabies epidemiology in Brazil. The detection of AgV3 in cases attributed to cats and dogs further suggests that domestic animals may act as bridge hosts between sylvatic and human transmission. These findings reinforce the need to strengthen wildlife exposure surveillance, ensure timely and equitable access to PEP, improve the completeness of SINAN records, and maintain high canine vaccination coverage as part of an integrated strategy to prevent human rabies deaths in an era of increasingly complex sylvatic transmission.

## SUPPLEMENTARY MATERIAL

SUPPLEMENTARY MATERIAL

## Data Availability

The complete anonymized dataset supporting the findings of this study is available from https://doi.org/10.48331/SCIELODATA.SBOSTJ
