# Supplementary material for: Temporal changes in rabies epidemiology in Brazil: an analysis of notifications of suspected and confirmed human rabies cases, 2010-2025
Source: Rev Inst Med Trop Sao Paulo. 2026 Sep 18;68:e60. doi: 10.1590/S1678-9946202668060 (PMC13588562; doi:10.1590/S1678-9946202668060)
Supplement: Supplementary file 1 [file 1678-9946-rimtsp-68-S1678-9946202668060-md1.pdf]

REVISTA DO INSTITUTO DE MEDICINA TROPICAL DE SÃO PAULO

JOURNAL OF THE SÃO PAULO INSTITUTE OF TROPICAL MEDICINE

SUPPLEMENTARY MATERIAL

https://doi.org/10.48331/SCIELODATA.SBOSTJ

Temporal changes in rabies epidemiology in Brazil: an analysis of notifications of suspected and confirmed human rabies cases, 2010–2025

João Vitor Engelhardt [1], Halana do Carmo Silva [2], Gabriel Campana do Nascimento [2], Arthur Moraes Lavinas [2], Beatriz Biancardi de Campos [2], Eduarda Scárdua Coser [2], Bruno Vidal Boscaglia [2], Elinara Ferreira Santos [2], Sarah Fernandes Teixeira [1], Mariana Rambaldi do Nascimento [1], Joamyr Victor Rossoni Júnior [1], Clairton Marcolongo-Pereira [1]

[1]Centro Universitário do Espírito Santo, Faculdade de Medicina, Programa de Pós-Graduação em Ciências da Saúde, Colatina, Espírito Santo, Brazil

[2]Centro Universitário do Espírito Santo, Faculdade de Medicina, Colatina, Espírito Santo, Brazil

Correspondence to: Clairton Marcolongo Pereira
Centro Universitário do Espírito Santo, Faculdade de Medicina, Programa de Pós-Graduação em Ciências da Saúde, Av. Fioravante Rossi, 2930, CEP 29703-855, Colatina, ES, Brazil
E-mail: cmpereira@unesc.br

Received: 8 June 2026
Accepted: 27 July 2026

Editor: Camila Malta Romano

Supplementary Table S1 - Annual number of human rabies notifications and confirmed cases in Brazil, 2010–2025, according to SINAN.

Year	Notifications	Confirmed cases
2010	77	3
2011	95	2
2012	78	5
2013	72	5
2014	68	0
2015	71	2
2016	111	2
2017	228	6
2018	174	11
2019	127	1
2020	148	2
2021	198	1
2022	7	2
2023	81	0
2024	112	2
2025	9	1
Total	1,482	46

Supplementary Table S2 - Distribution of human rabies notifications in Brazil according to age group and sex, 2010–2025, based on SINAN data.

Age group (years)	Female	Male	Unknown sex
0–4	52	80	0
5–9	74	92	0
10–14	52	87	0
15–19	44	52	0
20–29	76	105	1
30–39	92	127	0
40–49	86	98	1
50–59	63	78	0
≥ 60	110	110	0
Unknown age	1	0	1
Total	650	829	3

This is an open-access article distributed under the terms of the Creative Commons Attribution License.

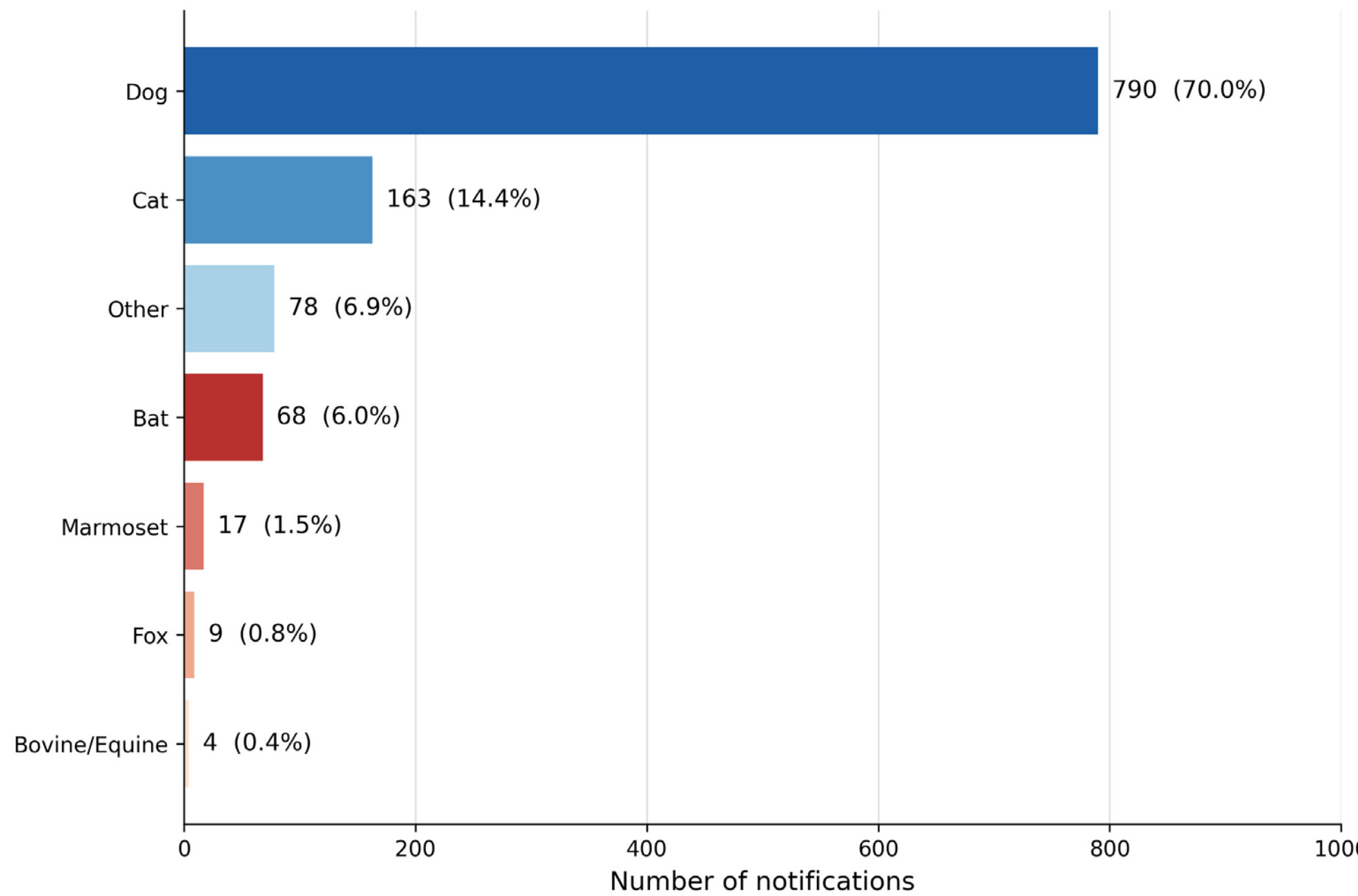

Supplementary Figure S1 – Human rabies notifications by aggressor species in Brazil, 2010–2025. Records with unknown species were excluded (valid species information for 1,204 of the 1,482 total notifications). Values indicate absolute counts and proportions relative to the total number of identified species.
